# Determinants of Health-Related Quality of Life in Patients with Chronic Kidney Disease: A Cross-Sectional Study

**DOI:** 10.3390/healthcare13101167

**Published:** 2025-05-16

**Authors:** Geetha Kandasamy, Thangamani Subramani, Mona Almanasef, Khalid Orayj, Eman Shorog, Asma M. Alshahrani, Tahani S. Alanazi, Sangeetha Balasubramanian

**Affiliations:** 1 Department of Clinical Pharmacy, College of Pharmacy, King Khalid University, Abha 62521, Saudi Arabia; glakshmi@kku.edu.sa (G.K.);; 2 Department of Pharmacy Practice, Grace College of Pharmacy, Palakkad 678004, India; 3 Department of Clinical Pharmacy, College of Pharmacy, Shaqra University, Dawadimi 11911, Saudi Arabia

**Keywords:** CKD, HRQoL, EQ-5D-3L, glomerular filtration rate, CKD stages

## Abstract

**Background:** Chronic kidney disease (CKD) significantly affects health-related quality of life (HRQoL), impacting physical and mental well-being. This study aimed to identify the key determinants influencing HRQoL among patients with CKD. **Methods:** A cross-sectional observational study was conducted from July 2022 to March 2023 at the Rajiv Gandhi Cooperative Multi-Specialty Hospital, Palakkad, Kerala, South India, including 154 patients diagnosed with CKD stages 3 to 5. Eligible participants were required to be at least 18 years of age and have a confirmed diagnosis of CKD, specifically stages 3 to 5, with prior treatment. CKD stages were defined according to the Kidney Disease: Improving Global Outcomes (KDIGO) 2012 guidelines, based on estimated glomerular filtration rate (eGFR) thresholds as follows: Stage 3 (eGFR 30–59 mL/min/1.73 m^2^), Stage 4 (eGFR 15–29 mL/min/1.73 m^2^), and Stage 5 (eGFR < 15 mL/min/1.73 m^2^). Participants were classified into stages based on their most recent stable eGFR value at the time of recruitment. HRQoL was assessed using the European Quality of Life-5 Dimensions-3 Levels (EQ-5D-3L) questionnaire. Chi-square, ANOVA, and multivariate regression were used to analyze associations with EQ-5D-3L domains. **Results:** Out of 154 participants, 68.8% were male, 91.6% were aged over 50 years, and 63.6% were from rural areas. Most had primary education (55.2%) and were unemployed, retired, or housewives (66.2%). As CKD progressed, comorbidities, particularly diabetes mellitus and coronary artery disease (CAD), increased, with Stage 5 showing the highest prevalence. Clinical markers showed significant declines in the glomerular filtration rate (GFR) (Stage 3: 49.16 ± 7.59, Stage 4: 22.37 ± 3.88, Stage 5: 8.79 ± 1.68) and hemoglobin (Stage 3: 10.45 ± 0.84, Stage 4: 8.88 ± 0.60, Stage 5: 7.12 ± 0.53) and an increase in serum creatinine (Stage 3: 1.72 ± 0.40, Stage 4: 3.21 ± 0.44, Stage 5: 7.05 ± 1.46). HRQoL assessments showed significant declines in mobility, self-care, usual activities, pain, and anxiety/depression with advancing CKD. Mobility issues increased from 61.2% in Stage 3 to 62.0% in Stage 5, with greater difficulties in self-care and usual activities at Stage 5. Pain and anxiety/depression worsened across stages. Multivariate analysis identified female gender, older age (≥50 years), lower education, unemployment, multiple comorbidities, smoking, lack of social support, and advanced CKD stages as significant factors linked to impaired HRQoL. CKD stage 5 (GFR < 29 mL/min/1.73 m^2^) and high serum creatinine (>1.2 mg/dL) were associated with significantly higher odds of impairment in all HRQoL domains. **Conclusions:** This study highlights that factors such as female gender, older age, lower education, unemployment, multiple comorbidities, smoking, advanced CKD stages, and high serum creatinine levels are associated with reduced quality of life in CKD patients. Conversely, social support acts as a protective factor. The findings emphasize the need for targeted interventions that address both medical care and psychosocial aspects, including lifestyle changes, patient education, mental health support, and community involvement, to improve CKD patients’ well-being.

## 1. Introduction

Chronic kidney disease (CKD) is defined by a glomerular filtration rate (GFR) lower than 60 mL/min/1.73 m^2^, albuminuria greater than 30 mg per 24 h, or ongoing evidence of kidney damage lasting for over three months [1]. CKD is more prevalent in low- and middle-income countries compared to high-income nations [2]. Although diabetes and hypertension are the primary causes of CKD globally, glomerulonephritis, infections, and environmental factors (NSAIDs, including pesticide exposure, the use of herbal medicines, and air pollution) also contribute significantly to CKD in regions such as Asia, sub-Saharan Africa, and other developing areas [3,4]. In India, approximately 17.2% of the population is affected by CKD, with 6% experiencing Stage 3 or more advanced stages of the disease. The increasing prevalence of CKD is primarily attributed to the rising incidence of risk factors such as diabetes and hypertension [5].

Quality of life (QoL), according to the World Health Organization (WHO), refers to an individual’s perception of their position in life within the context of the cultural and value systems they live in and in relation to their goals, expectations, standards, and concerns [6]. CKD has become a significant burden on global healthcare systems [7] and poses a substantial threat to patients’ QoL as the disease advances [8]. Health-related quality of life (HRQoL) describes how individuals or groups perceive their physical and mental well-being over a period of time. It is considered a key indicator for evaluating overall health and quality of life [9]. HRQoL plays a significant role in CKD management by impacting patients’ well-being, disease progression, and treatment decisions. Evidence demonstrates that individuals with CKD experience poorer HRQoL compared to the general population, with further deterioration as renal function declines. Even minor reductions in estimated glomerular filtration rate (eGFR) negatively affect HRQoL, particularly in older adults [10]. Poor QoL is a major concern for CKD patients, as it can negatively influence disease progression. Increased psychological distress, higher symptom burden, and diminished QoL are common among CKD patients, with the severity of these factors inversely correlated with GFR [11].

The negative impact on QoL is particularly pronounced in individuals with advanced kidney disease. Compared to other chronic conditions, utility-based QoL scores are lowest among patients with end-stage kidney disease (ESRD). QoL is a critical indicator for assessing disease burden, evaluating treatment efficacy, and predicting adverse outcomes. Identifying the factors influencing QoL provides valuable insights for nephrologists to improve patient care. In managing chronic diseases, healthcare professionals prioritize enhancing QoL alongside reducing mortality and morbidity [12]. Understanding the factors that adversely affect QoL in CKD patients is essential for implementing effective interventions. Studies have demonstrated that lower QoL scores are strongly associated with higher risks of mortality and hospitalization, surpassing traditional clinical parameters such as serum albumin levels [13]. Despite widespread evidence indicating lower QoL among CKD patients compared to healthy individuals, improving their life expectancy encompasses physical, psychological, and social dimensions of health, each comprising multiple components that can be perceived differently by patients, resulting in diverse QoL assessments [14].

An Indian study revealed that individuals with chronic kidney disease (CKD) experienced a significantly reduced health-related quality of life (HRQoL), with a progressive decline in HRQoL dimensions as the disease advanced to end-stage renal disease (ESRD) [15]. Additionally, diabetes and hypertension, the leading causes of ESRD, are highly prevalent in India. Despite improvements in health indicators, the country faces a high number of hemodialysis patients, emphasizing the need to assess HRQoL among CKD patients in regions with advanced healthcare infrastructure but a substantial disease burden [16].

Regular assessment of subjective well-being and functional status is recommended for patients with CKD and ESRD. These assessments, collectively referred to as HRQoL indicators, provide critical insights into patient care with disease management and also aid in understanding how various factors influence patients’ lives and allow comparisons between CKD patients and healthy individuals [15,17]. Hence, this study aims to identify the key determinants influencing HRQOL among patients with chronic kidney disease, with the goal of informing targeted interventions to improve patient outcomes.

## 2. Methods

### 2.1. Study Design

This cross-sectional observational study was conducted over a period of nine months (July 2022 to March 2023) at the Rajiv Gandhi Cooperative Multi-Specialty Hospital, a tertiary care center located in Palakkad, Kerala, South India. A total of 154 patients diagnosed with CKD were included in the study. The primary outcome variable was health-related quality of life (HRQoL), assessed using the EQ-5D-3L instrument. The predictor variables included sociodemographic characteristics (age, gender, education, employment, marital status, and residence), lifestyle behaviors (smoking, alcohol use, and social support), and clinical variables (CKD stage, comorbidities such as diabetes, hypertension, and coronary artery disease, blood pressure, hemoglobin level, serum creatinine, and GFR).

### 2.2. Population Size and Population Criteria

**Figure 1:**
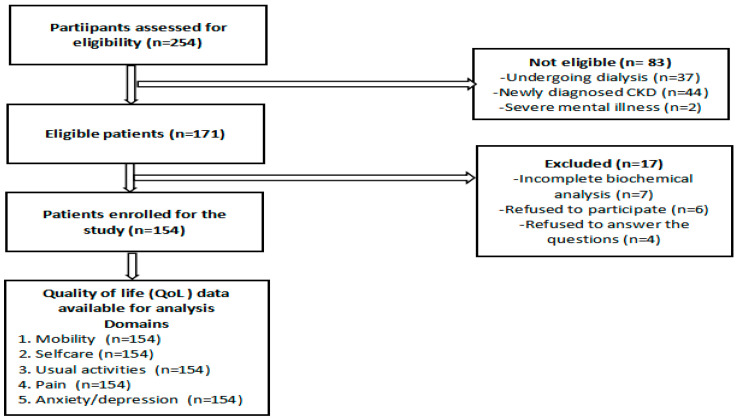
Participant flowchart.

Over the study period, approximately 254 patients visited the nephrology department, averaging 28–29 patients per month. The required sample size of 154 was calculated using the Raosoft sample size calculator, based on a 5% margin of error, 95% confidence level, and 50% response distribution. Participants were recruited consecutively based on inclusion criteria until the desired sample size was met. Eligible participants were required to be at least 18 years of age and have a confirmed diagnosis of CKD, specifically stages 3 to 5, with prior treatment. CKD stages were defined according to the Kidney Disease: Improving Global Outcomes (KDIGO) 2012 guidelines, based on estimated glomerular filtration rate (eGFR) thresholds as follows: Stage 3 (eGFR 30–59 mL/min/1.73 m^2^), Stage 4 (eGFR 15–29 mL/min/1.73 m^2^), and Stage 5 (eGFR < 15 mL/min/1.73 m^2^). Participants were classified into stages based on their most recent stable eGFR value at the time of recruitment. Exclusion criteria included individuals below 18 years of age, those with CKD Stages 1 or 2, patients undergoing dialysis or with a history of kidney transplantation, individuals with cognitive impairment or severe psychiatric illness, critically ill patients or those unable to communicate effectively, and those who declined to provide informed consent (Figure 1).

### 2.3. Data Collection Procedure

Informed consent was obtained from all participants before their inclusion in the study. A structured data collection form was used to record the following variables: Demographic factors: gender, age (≤50/>50 years), residence, educational status, employment status, and marital status; lifestyle factors: smoking status, alcohol intake, and social support; and clinical characteristics: blood pressure status, number and types of comorbidities (diabetes mellitus, hypertension, dyslipidemia, coronary artery disease, and others), and laboratory parameters such as GFR (mL/min/1.73 m^2^), hemoglobin (g/dL), and serum creatinine (mg/dL). HRQoL was assessed using the validated European Quality of Life-5 Dimensions-3 Levels (EQ-5D-3L) questionnaire. The questionnaire was administered through face-to-face interviews, and responses were directly entered into a Google Form to facilitate data extraction and analysis. The EQ-5D-3L questionnaire was translated into Malayalam, the local language, with assistance from subject matter experts. To ensure both linguistic accuracy and cultural appropriateness, a back-translation process was employed. This process involved translating the Malayalam version back into English by an independent translator, allowing for a comparison with the original English version to identify and resolve any discrepancies. Additionally, local healthcare professionals familiar with the study population reviewed the translation to ensure the language was clear, culturally relevant, and appropriate for patients with chronic kidney disease.

### 2.4. EQ-5D-3L

To assess HRQoL, the standardized tool used was the European Quality of Life 5-Dimension 3-Level (EQ-5D-3L) questionnaire [18,19]. This tool evaluates five key dimensions of QoL: mobility, self-care, usual activities, pain/discomfort, and anxiety/depression (5D). Each dimension includes a set of three response levels, allowing participants to self-assess the severity of their condition: Level 1: no problems; Level 2: some problems; Level 3: severe problems. Responses were used to generate a descriptive health profile for each participant. To ensure reliability, internal consistency was assessed using Cronbach’s alpha, which yielded a value of 0.8, indicating good reliability. In addition, the EQ-5D-3L is a widely validated tool with established validity in chronic disease populations, including patients with CKD.

### 2.5. Ethical Approval

The study was conducted in accordance with the ethical principles outlined in the Declaration of Helsinki. Written informed consent was obtained from all participants prior to enrollment. The study received approval from the Institutional Ethical Committee (Approval No. GCP/IEC/112B/2022) on 5 July 2022, prior to its commencement. Participant confidentiality was strictly maintained throughout the study, and all data were anonymized and used solely for research purposes.

### 2.6. Statistical Analysis

Data were analyzed using SPSS software, version 18. Chi-square tests were employed to assess associations between categorical variables, while an Analysis of Variance (ANOVA) was used to compare the means of continuous variables across categories. When ANOVA results were statistically significant, post hoc comparisons were conducted using Tukey’s Honestly Significant Difference (HSD) test. Additionally, multivariate logistic regression was performed to evaluate the relationship between independent variables (e.g., demographic, lifestyle, and clinical factors) and the EQ-5D-3L domains as dependent variables. A *p*-value of <0.05 was considered statistically significant. Each EQ-5D-3L domain was dichotomized for binary logistic regression: Level 1 (no problems) was grouped as one category, while Levels 2 and 3 (some problems and severe problems) were grouped as the other. Multicollinearity was assessed using the Variance Inflation Factor (VIF), and outliers were evaluated using standardized residuals.

## 3. Results

This prospective hospital-based study was conducted to assess the HRQoL among 154 patients aged 18 years or older with Stage 3 to 5 CKD, conducted over a nine-month period. Among the participants, 106 (68.8%) were male and 48 (31.2%) were female. The majority of patients (n = 141, 91.6%) were aged above 50 years, and 98 (63.6%) resided in rural areas. Regarding educational status, 85 patients (55.2%) had primary education, while 51 (33.1%) were illiterate. Most participants (n = 102, 66.2%) were unemployed, retired, or housewives, and 140 (90.9%) were married. Smoking and alcohol use were reported by 24 (15.6%) and 7 (4.5%) patients, respectively, while 30 (19.5%) reported having social support. Overall, the majority of patients were male, aged over 50 years, from rural areas, with low educational attainment, and were either unemployed or retired. Smoking, alcohol consumption, and social support were reported at relatively low rates across all CKD stages. No significant differences in sociodemographic characteristics were observed across CKD stages (*p* > 0.05) (Table 1).

Stage 3 patients showed a significantly higher proportion in the normal/prehypertensive blood pressure category (67.7%), whereas Stage 4 patients showed a greater prevalence of Stage 1/2 hypertension (57.1%). The number of comorbidities increased significantly with advancing CKD stages (*p* < 0.001). Stage 3 patients predominantly had one or two comorbidities, while the proportion of patients with three or more comorbidities was highest in Stage 5 (48.3%). Among specific comorbidities, diabetes mellitus showed a significantly higher proportion with CKD progression (*p* < 0.001), increasing from 12.9% in Stage 3 to 62.1% in Stage 5. Coronary artery disease (CAD) was also significantly more prevalent in Stage 5 (55.2%) compared to Stage 3 (24.2%) and Stage 4 (12.7%) (*p* < 0.001). Additionally, other comorbidities were more frequently reported among Stage 3 patients (*p* = 0.002). However, no significant differences were observed in the prevalence of hypertension (*p* > 0.05) and dyslipidemia (*p* = 0.688) across the CKD stages.

One-way ANOVA revealed significant differences in mean GFR, hemoglobin, and serum creatinine levels across CKD stages. GFR significantly declined with disease progression: Stage 3 (49.16 ± 7.59), Stage 4 (22.37 ± 3.88), and Stage 5 (8.79 ± 1.68) (*p* < 0.05). Hemoglobin levels also significantly decreased with advancing stages: Stage 3 (10.45 ± 0.84), Stage 4 (8.88 ± 0.60), and Stage 5 (7.12 ± 0.53) (*p* < 0.001). In contrast, serum creatinine levels increased significantly across the stages: Stage 3 (1.72 ± 0.40), Stage 4 (3.21 ± 0.44), and Stage 5 (7.05 ± 1.46) (*p* < 0.001). Tukey’s HSD post hoc test confirmed significant differences between all stages for these parameters (*p* < 0.05). Table 2 presents the clinical characteristics of patients with CKD Stages 3, 4, and 5.

Table 3 summarizes the distribution of CKD patients’ responses across the five EQ-5D-3L dimensions by CKD stages, revealing statistically significant differences across all domains. The proportion of patients reporting no problems in mobility significantly declined from Stage 3 at 38 (61.2%) to Stages 4 and 5 (20.6%), while moderate and severe mobility issues increased with disease progression (*p* = 0.0003). In terms of self-care, severe difficulties (Level 3) were reported by 17 (58.6%) patients in Stage 5, compared to only 1 (1.6%) in Stage 3 and 2 (3.1%) in Stage 4 (*p* < 0.001). Similarly, severe limitations in usual activities were observed in 18 (62.0%) Stage 5 patients, in contrast to 1 (1.6%) patient in Stage 3 (*p* < 0.001). The occurrence of severe pain/discomfort increased with disease severity, affecting 17 (58.6%) patients in Stage 5, while most Stage 3 patients reported no pain (*p* < 0.001). Anxiety/depression levels also showed a significant difference according to CKD stage, with severe symptoms reported by 6 (20.6%) Stage 5 patients and a consistent decline in patients reporting no anxiety/depression from Stage 3 to Stage 5 (*p* = 0.0005).

Table 4 presents the findings of the multivariate logistic regression analysis examining the association between sociodemographic variables, clinical factors, and EQ-5D-3L domains among patients with chronic kidney disease (CKD). Table 5 presents the model fit statistics for the logistic regression models across each of the five EQ-5D-3L domains. The analysis evaluates the impact on five domains: mobility, self-care, usual activities, pain, and anxiety/depression. Female patients showed a significantly higher odds ratio (OR) compared to males across all EQ-5D-3L domains. The odds of impaired mobility were 1.45 times higher among females (95% CI: 1.12–1.87, *p* = 0.002), while the odds of impaired self-care were 1.32 times higher (95% CI: 1.01–1.74, *p* = 0.045). Additionally, females had significantly higher odds of limitations in usual activities (OR = 1.28, 95% CI: 1.02–1.61, *p* = 0.038), pain (OR = 1.41, 95% CI: 1.10–1.81, *p* = 0.005), and anxiety/depression (OR = 1.38, 95% CI: 1.09–1.74, *p* = 0.009).

Patients aged ≥50 years exhibited significantly higher odds of impairment in all domains compared to those aged <50 years. The odds ratios ranged from 1.88 to 2.12, with *p* < 0.001 for all domains, indicating a strong association between older age and decreased quality of life. Illiterate patients had significantly higher odds of impairment in all domains when compared to those with a secondary or degree-level education. The highest odds ratio was observed for mobility (OR = 1.89, 95% CI: 1.42–2.51, *p* < 0.001). Similarly, primary education was also associated with a higher likelihood of impairment, with ORs ranging from 1.30 to 1.44 across different domains. Unemployed, retired, or housewife patients had significantly increased odds of impairment compared to employed patients. The odds of impaired mobility were 1.68 times higher (95% CI: 1.28–2.21, *p* < 0.001), while the odds for anxiety/depression were 1.53 times higher (95% CI: 1.17–2.00, *p* = 0.003).

Patients with three or more comorbidities had significantly higher odds of impaired quality of life across all domains compared to those with 0–2 comorbidities. For instance, the odds of impaired mobility were 2.05 times higher (95% CI: 1.53–2.74, *p* < 0.001). Smokers had significantly higher odds of impairment in all domains compared to non-smokers, with the highest odds observed for mobility (OR = 1.58, 95% CI: 1.19–2.10, *p* = 0.002). Alcohol consumption did not show a statistically significant association with any of the EQ-5D-3L domains, with *p*-values ranging from 0.600 to 0.873. Patients who lacked social support had significantly lower odds of impairment across all domains compared to those with support. The odds of impaired mobility were reduced (OR = 0.72, 95% CI: 0.54–0.95, *p* = 0.022), indicating a protective effect of social support. Patients in CKD stages 4 to 5 (GFR < 29 mL/min/1.73 m^2^) had significantly higher odds of impairment compared to those in stage 3 (GFR 30–59 mL/min/1.73 m^2^). The odds of impaired mobility were particularly high (OR = 2.45, 95% CI: 1.87–3.21, *p* < 0.001). Patients with serum creatinine levels > 1.2 mg/dL showed significantly higher odds of impairment across all domains compared to those with serum creatinine ≤ 1.2 mg/dL. The odds of impaired mobility were notably high (OR = 2.85, 95% CI: 2.08–3.90, *p* < 0.001).

## 4. Discussion

This cross-sectional hospital-based study aimed to assess the Health-Related Quality of Life (HRQoL) among 154 patients, aged 18 years or older, diagnosed with Stage 3 to 5 chronic kidney disease (CKD), over a nine-month period. CKD is a major global health concern and a serious non-communicable disease that has negative clinical and financial consequences [20]. Strategic planning for the prevention and management of CKD will benefit from more recent studies to ascertain the disease state, given the high prevalence of CKD and its expensive burden [21]. In comparison to female patients, male patients had a higher prevalence of CKD (67.7%). On the other hand, an individual patient data meta-analysis comprising 11 randomized controlled trials involving 1860 patients with CKD that employed angiotensin-converting enzyme inhibitors concluded that women may progress in renal illness at a faster rate than men [22]. In addition to causing structural and functional alterations, aging also raises the risk of kidney illnesses due to the immune system’s deterioration [23]. Smoking and alcohol consumption were significant risk factors for CKD among the study population, and through endothelial dysfunction, glomerulosclerosis, prothrombotic shift, oxidative stress, proinflammatory states, and tubular atrophy, smoking can raise the risk of CKD [24].

The study showed that hypertension was present in 57% of CKD patients. Hypertension is more common in CKD patients (80–85%) than in the general population (28.5%), according to a previous study [25]. In 57% of cases of hypertension, 43.5% of patients with chronic kidney disease received treatment for both Stage 1 and Stage 2 hypertension. Since blood pressure control prolongs the life expectancy of patients with renal disease and lessens structural and functional damage to nephrons, it is essential [26]. Patients with CKD frequently have treatment-resistant hypertension, elevated blood pressure at night, and masked hypertension, which are linked to various negative cardiovascular outcomes, including decreased eGFR, increased albuminuria, end-stage kidney disease progression, and hypertension-mediated organ damage [27].

As a common measure for assessing health-related outcomes, HRQOL is used to assess the influence of physical and psychological elements on an individual’s health [28]. According to our findings, QOL significantly declines over time as renal illness progresses (*p* < 0.05). Patients with CKD in Stages 4 and 5 reported greater moderate to extreme problems in all five dimensions, including mobility, self-care, usual activities, pain, and anxiety and depression. In the study, the most commonly affected domains among the study population were mobility (65.5%) and usual activities (62%), followed by self-care (58.6%), pain (58.6%), and anxiety and depression (55.1%). A prior study revealed that the highest proportion of participants (43%) reported experiencing pain, while 29% faced anxiety or depression, another 29% had reduced mobility, and 28% encountered difficulties in carrying out daily activities. Another study found that the most frequently affected domain was mobility (59.4%), which was followed by pain (47.8%) [29].

The findings of this study underscore the importance of the early identification and ongoing monitoring of risk factors for poor HRQoL in CKD patients, especially among older adults, women, the unemployed, those with low education, and those with multiple comorbidities. Integrating psychological and social assessments into routine care and implementing targeted interventions such as education programs, mental health support, and enhanced social services can improve patient well-being. Policymakers should prioritize expanding access to multidisciplinary care, particularly in underserved areas. Future research should assess the long-term impact of these interventions on HRQoL, adherence, and survival.

The implementation of targeted therapies and interventions to lower risk and improve protective factors for this vulnerable population of CKD patients depends on knowing about and understanding the major factors that may affect the overall and domain-specific quality of life. Furthermore, higher rates of mortality may be linked to a lower QoL [30,31]. Factors like female sex, age ≥ 50 years, illiterate and primary educational level, unemployed/retired/housewife patients, number of comorbidities (≥3), smoker, alcoholic, Stages 3 and 4, serum creatinine > 1.2 (mg/dL), and no social support were significantly associated poor QoL in all dimensions, and this finding was consistent with previous studies [32,33,34].

In our study, CKD patients who were older (>50 years) were significantly associated with poor QoL than those with ≤50 years. This finding aligns with an Australian study, which reported that younger individuals with chronic kidney disease (CKD) experienced significantly better quality of life compared to their older counterparts [35]. Chronic illnesses like diabetes, heart disease, lung disease, and hypertension are very common in the older population. When compared to the general population, those with less muscle mass engage in less physical activity. Additionally, cognitive impairment is more common in the elderly than in younger individuals [36]. The present study showed that women with CKD had lower QoL compared to men, and a previous study in this area indicated the same finding. Cultural differences provide support for the current study’s findings. Our society is conservative and dominated by men, with firmly established social roles. Despite having a chronic illness, men reported higher levels of social support and quality of life than women, as they are valued more than women in our society [37].

This study demonstrates that CKD patients without social support were significantly associated with poor QoL. Social isolation has been associated with lower self-rated health, greater mortality, higher rates of depression, and negative health behaviors like poor diet and treatment noncompliance [38]. CKD patients who do not have a spouse or partner (e.g., single, widowed, or divorced) are associated with poorer overall quality of life (QoL) and problems with mobility, daily activities, and anxiety or depression. This suggests that social support, such as focus groups, virtual or online support, and patient/community navigators, may improve QoL and the treatment of serious illnesses [39,40].

The current study further demonstrated that patients with CKD who are unemployed, retired, or housewives, as well as those with a low educational level, have a considerably lower HRQoL [41,42]. It is also known that gender and age have an impact on employment rates. According to the findings, patients without employment had a lower QoL than those with occupations. Their professional prospects were negatively affected by the substantial effects on their symptoms, disease burden, and physical and mental health [43]. Better mobility, financial independence, and fewer restrictions on daily activities might have improved these patients’ QoL [44]. Furthermore, the study found that people with less education had a lower quality of life across all dimensions. According to previous studies, education is linked to a high quality of life for people with CKD [45,46]. Patients with higher levels of education have better access to information about their illness, better financial circumstances, and healthier health-seeking habits [47].

In patients with CKD, having three or more comorbidities was significantly associated with a lower quality of life. These findings were in line with other studies, and patients’ disease burden worsens as the number of comorbidities increases [28,47,48]. In addition to the clinical burden of multiple comorbidities, the findings underscore the need for a more holistic approach in managing patients with CKD. A purely biomedical perspective may overlook important psychosocial factors that contribute to reduced quality of life [28,47,48]. Adopting a biopsychosocial model that considers the psychological, social, and environmental contexts alongside medical factors can significantly enhance patient outcomes. In India, access to multidisciplinary care for CKD patients remains limited, with such services typically available only in large tertiary care hospitals. Many patients in rural or resource-limited settings lack access to coordinated care involving nephrologists, dietitians, mental health professionals, and social workers. Given the complex and multifaceted nature of CKD and its comorbidities, we advocate for the development and implementation of integrated, multidisciplinary service models across healthcare settings in India. Such an approach could address not only the clinical but also the psychological and social needs of CKD patients, ultimately improving their health-related quality of life. The detrimental clinical and financial effects of CKD are significantly influenced by comorbidity. It is evident that “concordant” comorbid illnesses, including diabetes, heart failure, and hypertension, add to the excessive burden of comorbidity among CKD patients [49].

This study is important as it offers a comprehensive assessment of factors affecting HRQoL in Stage 3 to 5 CKD patients in India, where such data are scarce. It identifies key sociodemographic, clinical, and psychosocial determinants, including age, gender, education, employment, comorbidities, and social support, that influence QoL. By addressing a critical knowledge gap, the study supports the development of targeted, patient-centered interventions and highlights the need to integrate a biopsychosocial model into CKD care, particularly in resource-limited settings.

The study suggests actionable strategies to improve HRQoL in CKD patients, including implementing multidisciplinary care models, targeting high-risk groups with tailored interventions, strengthening social support systems, promoting patient education to boost health literacy and adherence, and advocating for expanded access to integrated care in rural and underserved areas.

### Limitations

The present study has certain limitations. First, it employed a cross-sectional design, which limits the ability to assess changes in quality of life (QoL) over time and establish causal relationships. Second, although the EQ-5D-3L is valuable for general psychometric comparisons across various diseases, it may lack sensitivity in detecting subtle QoL changes specific to chronic kidney disease (CKD), potentially underestimating the effects of financial hardship. Future research should include longitudinal studies to track QoL changes over time and explore causal pathways. Additionally, qualitative studies using approaches such as thematic analysis or interpretative phenomenological analysis (IPA) could provide deeper insights into the lived experiences and psychosocial factors that influence HRQoL in individuals with CKD, particularly in different cultural and socioeconomic contexts.

## 5. Conclusions

The findings from this study highlight that factors such as female gender, older age, lower educational attainment, unemployment, multiple comorbidities, smoking, advanced CKD stages, and elevated serum creatinine levels are significantly associated with a reduced quality of life among CKD patients. Conversely, social support acts as a protective factor, helping to mitigate the negative impact on QoL. These results emphasize the importance of implementing targeted interventions that address both medical and psychosocial aspects, including lifestyle changes, patient education, mental health support, and community involvement, to enhance the overall well-being of CKD patients.

## Figures and Tables

**Table 1:** Relationship between sociodemographic characteristics of the study population and different stages of chronic kidney disease (CKD).

Variables	Category	Stage 3 CKD (n = 62)	Stage 4 CKD (n = 63)	Stage 5 CKD (n = 29)	*p*-Value
Gender	Male	42 (67.7)	45 (71.4)	19 (65.52)	0.82
	Female	20 (32.3)	18 (28.6)	10 (34.48)	
Age in years	≤50 years	6 (9.7)	4 (6.3)	3 (10.34)	0.73
	>50 years	56 (90.3)	59 (93.7)	26 (89.66)	
Residence	Rural	37 (59.7)	43 (68.3)	18 (62.07)	0.59
	Urban	25 (40.3)	20 (31.7)	11 (37.93)	
Educational status	Illiterate	22 (35.5)	21 (33.8)	8 (27.59)	0.78
	Primary	35 (56.5)	33 (52.4)	17 (58.62)	
	Secondary/Degree	5 (8.1)	9 (14.3)	4 (13.79)	
Employment status	Employed	25 (40.3)	18 (28.6)	9 (31.03)	0.35
	Unemployed/retired/Housewife	37 (59.7)	45 (71.4)	20 (68.97)	
Marital Status	Married	58 (93.5)	55 (87.3)	27 (93.10)	0.43
	Unmarried/widowed	4 (6.5)	8 (12.7)	2 (6.90)	
Smoking	Yes	7 (11.3)	12 (19)	5 (17.24)	0.47
	No	55 (88.7)	51(81)	24 (82.76)	
Alcohol intake	Yes	5 (8.1)	1(1.6)	1 (3.45)	0.21
	No	57 (91.9)	62 (98.4)	28 (96.55)	
Social support	Yes	9 (14.5)	14 (22.2)	07 (24.14)	0.43
	No	53 (85.5)	49 (77.8)	22 (75.86)	

*p* < 0.05 is considered significant.

**Table 2:** Clinical characteristics of study population across different stages of chronic kidney disease (CKD).

Variables	Stage 3 CKD (n = 62)	Stage 4 CKD (n = 63)	Stage 5 CKD (n = 29)	*p*-Value
**Blood Pressure**				0.015 *
Normal/Prehypertension	42 (67.74)	27 (42.86)	18 (62.07)	
Stage 1/Stage 2	20 (32.26)	36 (57.14)	11 (37.93)	
**Number of comorbidities**				0.0001 *
1	29 (46.77)	25 (39.68)	3 (10.34)	
2	23 (37.10)	17 (26.98)	7 (24.14)	
≥3	8 (12.90)	11 (17.46)	14 (48.28)	
Nil	2 (3.23)	10 (15.87)	5 (17.24)	
**Specific comorbidities**				
**Diabetes Mellitus**				0.001 *
Yes	8 (12.90)	19 (30.16)	18 (62.07)	
No	54 (87.10)	44 (69.84)	11 (37.93)	
**Hypertension**				0.169
Yes	34 (54.84)	41 (65.08)	13 (44.83)	
No	28 (45.16)	22 (34.92)	16 (55.17)	
**Dyslipidemia**				0.688
Yes	14 (22.58)	16 (25.40)	9 (31.03)	
No	48 (77.42)	47 (74.60)	20 (68.97)	
**CAD**				0.00007 *
Yes	15 (24.19)	8 (12.70)	16 (55.17)	
No	47 (75.81)	55 (87.30)	13 (44.83)	
**Others**				0.002 *
Yes	40 (64.52)	21 (33.33)	13 (44.83)	
No	22 (35.48)	42 (66.67)	16 (55.17)	
**GFR (mL/min/1.73 m^2^)**	49.16 ± 7.59	22.37 ± 3.88	8.79 ± 1.68	<0.05 *
**Hb (gm/dL)**	10.45 ± 0.84	8.88 ± 0.60	7.12 ± 0.53	<0.001 *
**Serum Creatinine (mg/dL)**	1.72 ± 0.40	3.21 ± 0.44	7.05 ± 1.46	<0.001 *

CAD—coronary artery disease. GFR—glomerular filtration rate. Hb—hemoglobin. Post hoc analysis (Tukey’s HSD). * indicates Significance *p* < 0.05.

**Table 3:** Distribution of responses of CKD patients with different stages in five dimensions by using EQ 5D 3L Questionnaire.

Dimension	Level	Stage 3 (n = 62)	Stage 4 (n = 63)	Stage 5 (n = 29)	*p*-Value
**Mobility**	1	38 (61.2)	25 (39.6)	06 (20.6)	0.0003 *
	2	23 (37.0)	37 (58.7)	19 (65.5)	
	3	1 (1.6)	1 (1.6)	4 (13.7)	
**Selfcare**	1	48 (77.4)	34 (53.9)	9 (31.03)	0.001 *
	2	13 (20.9)	27 (42.2)	3 (10.3)	
	3	1 (1.6)	2 (3.1)	17 (58.6)	
**Usual activities**	1	24 (39.7)	28 (44.4)	8 (27.5)	0.001 *
	2	37 (59.6)	33 (52.3)	3 (10.3)	
	3	1 (1.6)	2 (3.1)	18 (62.0)	
**Pain/Discomfort**	1	35 (58.4)	20 (31.7)	6 (20.6)	0.001 *
	2	26 (41.9)	35 (55.5)	6 (20.6)	
	3	1 (1.6)	8 (12.6)	17 (58.6)	
**Anxiety/depression**	1	41 (66.1)	30 (47.6)	7 (24.1)	0.0005 *
	2	20 (32.2)	29 (46.0)	16 (55.1)	
	3	1 (1.6)	4 (6.34)	6 (20.6)	

*p* < 0.01 and * *p* < 0.05 indicate statistical significance.

**Table 4:** Multivariate binary logistic regression analysis showing the association between sociodemographic and clinical factors and EQ-5D-3L domains (dichotomized) among patients with chronic kidney disease.

Parameters	Mobility			Self-Care			Usual Activities			Pain			Anxiety/Depression		
	OR Ratio	CI	*p* Value	OR ratio	CI	*p* Value	OR Ratio	CI	*p* Value	OR Ratio	CI	*p* Value	OR Ratio	CI	*p* Value
**Gender**															
Male (n = 106)	Reference														
Female (n = 48)	1.45	1.12–1.87	0.002	1.32	1.01–1.74	0.045	1.28	1.02–1.61	0.038	1.41	1.10–1.81	0.005	1.38	1.09–1.74	0.009
**Age group**															
<50 years (n = 13)	Reference														
≥50years (n = 141)	2.12	1.56-2.89	<0.001	1.95	1.45–2.61	<0.001	2.07	1.53–2.81	<0.001	1.88	1.41–2.52	<0.001	1.92	1.44–2.55	<0.001
**Education**															
Illiterate (n = 51)	1.89	1.42–2.51	<0.001	1.75	1.32–2.33	<0.001	1.82	1.38–2.41	<0.001	1.68	1.26–2.24	<0.001	1.73	1.30–2.31	<0.001
Primary (n = 85)	1.44	1.11–1.86	0.006	1.32	1.01–1.72	0.042	1.37	1.05–1.79	0.020	1.30	1.01–1.68	0.044	1.33	1.03–1.72	0.036
Secondary/degree (n = 18)	Reference														
**Employment status**															
Unemployed/retired/Housewife (n = 102)	1.68	1.28–2.21	<0.001	1.55	1.18–2.05	0.002	1.60	1.22–2.10	<0.001	1.50	1.14–1.98	0.004	1.53	1.17–2.00	0.003
Employed (n = 52)	Reference														
**No. of comorbidities**															
≥3 (n = 32)	2.05	1.53–2.74	<0.001	1.89	1.41–2.54	<0.001	2.00	1.49–2.69	<0.001	1.85	1.38–2.48	<0.001	1.90	1.42–2.55	<0.001
0–2 (n = 122)	Reference														
**Smoking**															
No (n = 120)	Reference														
Yes (n = 24)	1.58	1.19–2.10	0.002	1.45	1.09–1.94	0.010	1.52	1.14–2.03	0.006	1.39	1.05–1.84	0.021	1.47	1.12–1.93	0.008
**Alcohol intake**															
Yes (n = 7)	0.95	0.52–1.75	0.873	0.89	0.49–1.62	0.720	0.92	0.50–1.69	0.800	0.85	0.46–1.58	0.600	0.88	0.48–1.63	0.730
No (n = 147)	Reference														
**Social Support**															
Yes (n = 30)	Reference														
No (n = 124)	0.72	0.54–0.95	0.022	0.68	0.51–0.91	0.010	0.70	0.52–0.94	0.018	0.66	0.49–0.90	0.009	0.69	0.51–0.93	0.015
**Stages of CKD GFR (mL/min/1.73 m^2^)**															
Stage 3 (n = 62) GFR 30-59	Reference														
Stage 4 to 5 (n = 92) (GFR less than 29)	2.45	1.87–3.21	<0.001	2.32	1.76–3.07	<0.001	2.40	1.82–3.15	<0.001	2.20	1.68–2.89	<0.001	2.28	1.74–2.99	<0.001
**Serum Creatinine**															
≤1.2 (mg/dL) (n = 11)	Reference														
>1.2 (mg/dL) (n = 143)	2.85	2.08–3.90	<0.001	2.62	1.91–3.60	<0.001	2.78	2.02–3.83	<0.001	2.50	1.81–3.46	<0.001	2.65	1.92–3.65	<0.001

OR—Odds Ratio. CI—Confidence Interval.

**Table 5:** Model fit Summary for each domain.

Domain	−2 Log Likelihood	Hosmer–Lemeshow χ^2^ (*p*-Value)
Mobility	172.45	5.72 (*p* = 0.573)
Self-care	190.88	6.29 (*p* = 0.505)
Usual Activities	185.12	4.61 (*p* = 0.710)
Pain/Discomfort	179.67	7.03 (*p* = 0.532)
Anxiety/Depression	187.90	6.82 (*p* = 0.447)

## Data Availability

The original contributions presented in the study are included in the article; further inquiries can be directed to the corresponding author.
